# Induction of subtle blood-brain barrier dysfunction using preclinical diagnostic ultrasound combined with microbubbles

**DOI:** 10.1186/s12987-026-00820-7

**Published:** 2026-06-02

**Authors:** Shakira A. van der Panne, Isabella Z. Koster, Anita E. Grootemaat, Nicole N. van der Wel, Mario G. Ries, Helga E. de Vries, Louise van der Weerd, Gustav J. Strijkers, Erik N. T. P. Bakker

**Affiliations:** 1 Department of Biomedical Engineering and Physics, Amsterdam University Medical Center, Amsterdam, The Netherlands; 2 Amsterdam Neuroscience, Amsterdam University Medical Center, Amsterdam, The Netherlands; 3 Amsterdam Cardiovascular Sciences, Amsterdam University Medical Center, Amsterdam, The Netherlands; 4 Electron Microscopy Center Amsterdam, Amsterdam University Medical Center, Amsterdam, The Netherlands; 5 Center for Imaging Sciences, University Medical Center Utrecht, Utrecht, The Netherlands; 6 Department of Molecular Cell Biology and Immunology, Amsterdam University Medical Center, Amsterdam, The Netherlands; 7 Department of Radiology, Leiden University Medical Center, Leiden, The Netherlands; 8 Department of Human Genetics, Leiden University Medical Center, Leiden, The Netherlands

**Keywords:** Blood-brain barrier, Power Doppler ultrasound, Microbubbles, Immunoglobulin, Leakage, Animal mouse model, Preclinical

## Abstract

**Background:**

The blood–brain barrier (BBB) plays a critical role in maintaining brain homeostasis by tightly regulating molecular transport. However, its integrity is often compromised with aging and in neurodegenerative diseases, contributing to disease pathology. Studying the biological consequences of BBB dysfunction independent of concomitant pathology remains challenging, largely due to the absence of reliable and inducible animal models that avoid unintended side effects such as osmotic effects, neuroinflammation, or vascular damage. In this study, we evaluated the use of Power Doppler ultrasound (PDUS) combined with microbubbles to induce widespread, bilateral BBB opening in the mouse brain.

**Methods:**

Mice received intravenous infusions of SonoMAC microbubbles during transcranial PDUS application. BBB permeability was assessed via Evans Blue dye extravasation and immunofluorescence analysis of extravasated immunoglobulins. Vessel integrity was evaluated at the ultrastructural level using transmission electron microscopy (TEM).

**Results:**

PDUS combined with microbubbles successfully induced widespread BBB opening, as evidenced by diffuse Evans Blue staining and immunoglobulin extravasation in coronal sections. Immunoglobulin leakage was detected in all analyzed brain regions, with lower levels in white matter, likely reflecting its lower vascular density. Leakage appeared to primarily originate from capillaries while TEM analysis revealed no overt vascular damage.

**Conclusions:**

These findings support PDUS with microbubbles as a non-destructive, reproducible method to model widespread BBB dysfunction. This approach offers an in vivo platform to study BBB-related pathophysiological processes such as impaired clearance, protein aggregation, and neurotoxicity, as well as for investigation of therapeutic delivery to the brain parenchyma.

**Supplementary Information:**

The online version contains supplementary material available at 10.1186/s12987-026-00820-7.

## Background

The blood-brain barrier (BBB) is a highly specialized structural and functional interface that protects the brain from intrusion by potentially harmful substances. At the same time, it contributes to maintaining central nervous system homeostasis by regulating the selective transport of nutrients into the brain and the removal of waste products. The BBB is formed by cerebral capillary endothelial cells (ECs) and tight junctions which together reduce the paracellular movement of ions and other hydrophilic solutes [1, 2]. The BBB also tightly regulates transcellular transport through mechanisms such as passive diffusion, receptor-mediated and adsorptive-mediated transcytosis, and carrier-mediated transport [1]. This barrier function is further maintained by the interaction of the ECs with neighboring cell types including pericytes, astrocytes, neurons, and microglia which together form a dynamic cellular network called the neurovascular unit [1, 3].

BBB dysfunction is a hallmark of aging and several neurodegenerative diseases including Alzheimer’s disease, Parkinson’s disease, Huntington disease, and amyotrophic lateral sclerosis [2, 4]. This dysfunction involves structural or functional impairments that result in a loss of barrier function, ultimately leading to a widespread increase in permeability [3]. While BBB breakdown has been correlated with these disorders, its causal role in disease pathogenesis remains difficult to establish. Transgenic animal models frequently display BBB dysfunction with overlapping disease-related processes, making it difficult to distinguish causal mechanisms. In contrast, inducible models opening the BBB provide a controlled approach to study the specific effects of BBB disruption in the absence of broader pathological changes. Several approaches have been developed to induce BBB opening, such as mannitol infusion [5], lipopolysaccharide exposure [6], photodynamic therapy [7], or photothrombosis using Rose Bengal [8]. However, these approaches often rely on inducing osmotic stress, inflammation, or vascular injury, which can confound results by triggering biological effects unrelated to BBB permeability itself [5–8]. A promising alternative is sonopermeation, a technique that employs ultrasound (US) in combination with microbubbles (MBs) to increase the permeability of biological barriers through acoustic cavitation [9, 10]. Unlike osmotic or inflammatory methods, sonopermeation acts through mechanical means to transiently disrupt the BBB. A particularly effective and emerging form of this technique is focused ultrasound (FUS) combined with MBs, which has been shown to induce localized, reversible, and safe BBB opening without causing overt vascular damage as analyzed by hematoxylin and eosin staining of erythrocyte extravasations [11, 12]. Despite these advantages, FUS is limited by its focal nature and is therefore less suitable for modelling diffuse or global BBB disruption. Therefore, we propose to combine MBs with Power Doppler ultrasound (PDUS) as a noninvasive method for inducing broader BBB opening. Unlike FUS, diagnostic US transducers use linear or phased ultrasound beams that can target larger brain regions. Although the higher frequencies and non-focused energy associated with diagnostic US applications such as PDUS reduce cavitation likelihood, optimizing US and MB parameters may overcome these limitations [13–15]. Prior studies have demonstrated the feasibility of BBB opening with diagnostic US and MBs, primarily in the context of drug delivery or tumor models. However, sonopermeation was either conducted in vitro, limited to small or unilateral brain regions, or lacked thorough assessment of BBB permeability across the whole brain, particularly for large macromolecules such as blood-derived proteins [9, 16–21]. In neurological disorders characterized by BBB dysfunction such as Alzheimer’s disease, these proteins are frequently found within brain tissue, making them especially relevant for clinically meaningful investigation [22, 23].

This study investigated the potential of PDUS, in combination with poly(butyl cyanoacrylate) (PBCA)-based MBs, to induce widespread BBB opening in mice without causing additional vascular damage. The goal was to develop this technique as a novel and controllable animal model for studying clinically relevant BBB dysfunction. BBB disruption was assessed macroscopically via Evans blue (EB) extravasation, and semi-quantified at the microscopic level through immunofluorescence (IF) detection of endogenous immunoglobulins (Igs). The spatial distribution of leakage and the associated vessel types were analyzed. Additional characterization included quantification of leakage hotspot size and identification of the cell types that showed interaction with extravasated Igs. Additionally, potential vascular damage was evaluated using transmission electron microscopy (TEM) to assess EC integrity at high resolution. Unlike conventional hematoxylin and eosin staining which primarily detects erythrocyte extravasation and is thus limited to identifying major vascular disruptions, TEM enables the detection of subtle ultrastructural changes, allowing for a more detailed and sensitive assessment of vascular integrity. The results of this study showed that this method provides a controlled and reproducible model for increased BBB permeability which will facilitate studies on its consequences in both healthy brain tissue and in the context of neurological diseases.

## Methods

### Animals

All animal experiments were approved by the Animal Ethics Committee of the University of Amsterdam and conducted in accordance with the ARRIVE guidelines and the European Union guidelines for laboratory animal welfare (Directive 2010/63/EU). Mice were group-housed under a 12 h light/dark cycle with ad libitum access to food and water and acclimatized for at least one week prior to the start of experiments. Twenty-six C57BL/6JOlaHsd mice (Envigo RMS, the Netherlands) aged 13–23 weeks were used. Of these, 18 mice were included in the IF experiments (*n* = 8 controls, *n* = 10 intervention) and 8 in the TEM experiments (*n* = 4 controls, *n* = 4 intervention). No formal power calculation was performed for the IF experiments. The sample size was informed by variability observed in pilot data, findings from similar published studies, and the need to account for potential data loss. Since the intervention group involved successful induction of a condition (BBB opening to generate a dysfunction model), the sample size was determined on practical grounds to ensure adequate representation of the expected variability. For TEM experiments, no sample size calculation was applicable as the experiments were intended for qualitative assessment of ultrastructural changes. Within each experimental set, individual animals were assigned to control or intervention groups in a pseudo-random manner, based on practical considerations. Allocation was balanced to maintain equal numbers of males and females across groups (*n* = 13 males, *n* = 13 females overall), to avoid individual housing, and by prioritizing older animals first. No formal randomization sequence (e.g., computer-generated allocation) was used. Animals with unsuccessful tail vein cannulation were excluded. Blinding was not possible during in vivo procedures, as the researcher needed to know group allocation to deliver the appropriate treatment. For subsequent IF and TEM analyses, animals were coded so that quantifications were performed blinded to group allocation, despite the same researcher performing both phases.

### BBB opening procedure

**Fig. 1:**
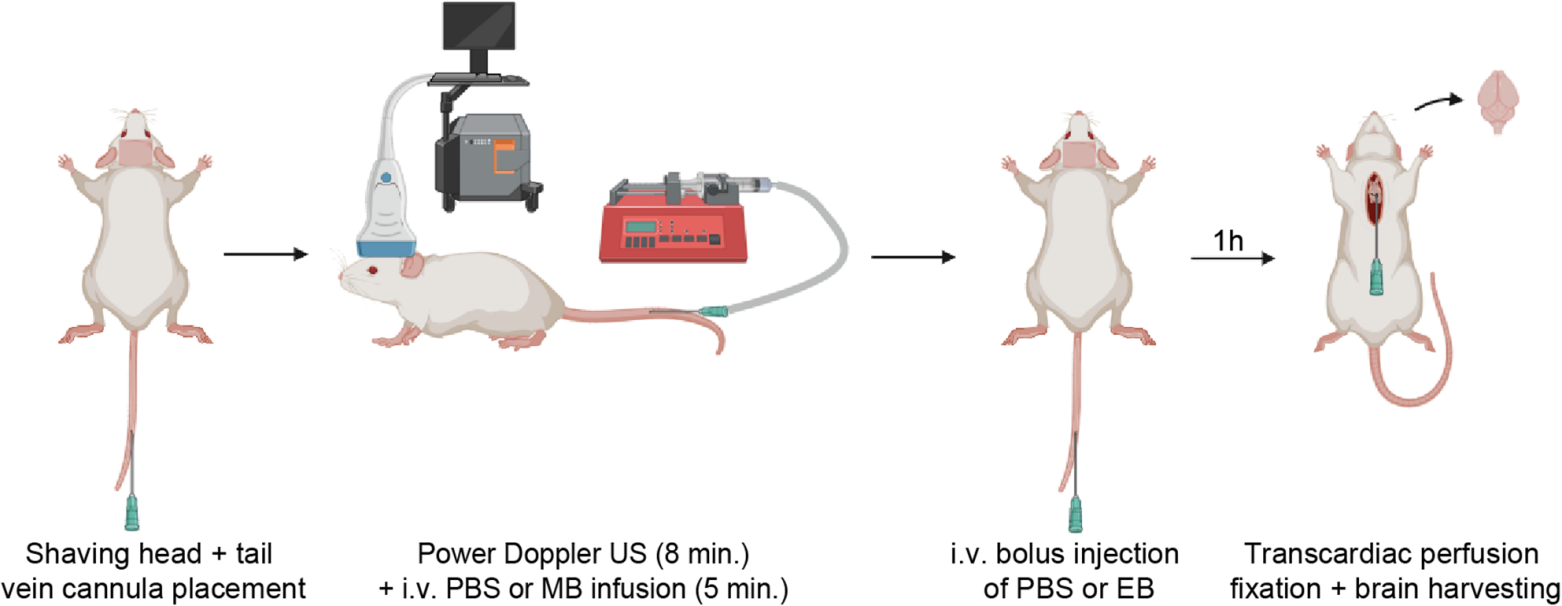
Schematic overview of experimental workflow of the BBB opening procedure. The whole procedure was conducted under 2% isoflurane anesthesia in 1:1 air: O_2_. Image created using BioRender.com

A schematic overview of the experimental workflow of the BBB opening procedure is presented in Fig. 1.

### Animal preparation

Animals were weighed and anesthetized using isoflurane (4% induction, 2% maintenance) in a 1:1 mixture of air:O_2_ in an induction chamber, followed by a nose cone for the rest of the experiment. The animals were placed on a heating pad in combination with a heating lamp, and a rectal thermal probe was inserted to monitor and maintain body temperature (37.2˚C ± 1˚C). Ocular lubricant (Duratears, Alcon) was applied to keep the eyes hydrated and the hair was removed from the head by a rodent shaver and depilatory cream (Veet). For placement of the lateral tail vein cannula, the animal was placed in lateral recumbency for access to the tail vein, and the tail was heated using the heating lamp for easy insertion. The 29G needle of an insulin syringe (0.33 × 12.7 mm, Microfine, Embecta) was broken off and connected with superglue to silicone tubing (0.012” ID x 0.024” OD, Sani-Tech Ultra-C-012–0 F, Hitma). The needle was inserted in the lateral tail vein and held into place with superglue.

### BBB opening

For PDUS application, the preclinical Vevo^®^ 3100 Imaging System (FUJIFILM VisualSonics Inc.) was used in combination with an MX201 linear array transducer (128 elements, ultra-high frequency, 22 MHz central frequency). The animal was placed in a prone position and warmed ultrasound gel was applied to the head. The transducer was placed perpendicular to the skull surface (ultrasound field = 12 × 8 mm) around 0 mm Bregma using B-mode imaging.

A 1 mL syringe filled with either PBS (control group) or lyophilized PBCA MBs (intervention group; SonoMAC-r, SonoMAC GmbH; size range = 1–5 μm) in a concentration of 1 × 10^9^ MBs/mL was connected to the silicone tubing of the tail vein cannula. The syringe containing the PBS or MBs was placed in an infusion pump, and 4 µL/gram of body weight was infused over a period of 5 min. At the start of infusion, an 8-min static US sonication was applied in power Doppler mode. Acoustic excitation was achieved using a Doppler imaging sequence with an image frame rate of 40 Hz, whereby each image consisted of one transducer element firing two 1.5-cycle pulses at 12.5 MHz with a pulse repetition frequency of 50 kHz. Not all transducer elements contribute equally to brain exposure (~ 50% uncoupled due to lateral positioning and cranial geometry), and each element generates a sinc-shaped far-field pressure profile. Cranial attenuation was accounted for at 14.9 dB at 12.5 MHz. Each voxel in the brain received a 4 ms burst pulse, consisting of 100 equally spaced 1.5-cycle pulses at 12.5 MHz, each with a peak-negative pressure of 390 kPa. This corresponds to a peak mechanical index of 0.11 and a duty cycle of 0.048% (see Supplementary Figure S1 for a schematic of the burst pulse train, and Supplementary Methods for a detailed description of the performed US parameter measurements).

After the sonication and MB infusion, a bolus injection of either 2% EB in PBS (for TEM experiments) or PBS (for IF experiments) was given via the tail vein cannula in a total volume of 4 µL/gram of body weight.

### Transcardiac perfusion fixation

After 1 h, the animals received a 20 µL intraperitoneal injection of undiluted heparin (5000 IU/mL, LEO) and were sacrificed using a transcardiac perfusion fixation in combination with an overdose of isoflurane (5%, 1:1 air: O_2_). Four mice of the IF experiment set (*n* = 2 controls and *n* = 2 intervention) received a 1 µL/gram of body weight bolus injection of 1 µg/µL lectin (lectin from *lycopersicon esculentum* (tomato) FITC, Sigma-Aldrich, L0401) via the tail vein cannula prior to the perfusion fixation in order to visualize the vasculature. The thorax was opened to expose the heart and a needle was inserted into the left ventricle. The right atrium was punctured and 10 mL of PBS was perfused to drain the blood from the tissue, followed by perfusion of 10 mL with either 4% paraformaldehyde (PFA) (for IF experiments) or 3% PFA + 3% glutaraldehyde (GA) (for TEM experiments; Electron Microscopy Sciences, 16538-07). After perfusion, the brain was removed from the skull. For IF experiments, the brain was placed in 4% PFA for 1 day at 4˚C, followed by cryoprotection for 2 days in 30% sucrose solution at 4˚C and subsequent storage at -80˚C until further use. For TEM experiments, the mid-cortical area between the anterior and posterior regions was directly cut into 1–2 mm cubes and stored in 3% PFA/3% GA.

### Transmission electron microscopy

The samples were fixed in 3% PFA + 3% GA for 1 day at room temperature followed by 6 days at 4˚C. For embedding, samples were post-fixed with 1% osmium tetroxide (OsO₄, Electron Microscopy Sciences, Hatfield, PA, USA) for 1 h. They were then dehydrated through a graded ethanol series (70% to 100%), followed by incubation in 1,2-propylene oxide (Sigma-Aldrich, Merck, Darmstadt, Germany). Infiltration was performed using a 1:1 and subsequently a 1:2 ratio of 1,2-propylene oxide to Epon resin (LX-112, Ladd Research, Williston, VT, USA), after which samples were embedded in pure Epon. Ultrathin 60-nm-thick sections were cut using a diamond knife (Diatome) on a Leica Ultracut UC7 ultramicrotome, and collected on Formvar-coated copper grids (Electron Microscopy Sciences, FF100H-Cu-50). Sections were counterstained with uranyl acetate and lead citrate (Electron Microscopy Sciences, 22410), and imaged using a Tecnai T12 transmission electron microscope equipped with a Xarosa camera and Radius software (EMSIS). Micrographs were acquired from all blood vessels visible within the tissue sections. Each vessel was visually examined to confirm the integrity of the endothelial lining and to assess the presence of extravasated erythrocytes.

### Immunofluorescence

Frozen brains were sectioned in 50-µm-thick coronal sections using a cryomicrotome (Thermo Cryostar NX70 Cryostat). Sections were stored at -20˚C in a 48-wells plate containing cryoprotectant solution (30% sucrose and 30% ethylene glycol in PBS) until further use.

For all intervention and control animals, free floating sections taken at ~-1.25 mm Bregma were used due to its central location in the brain and visibility of the hippocampal structures for analysis of regional differences in leakage. After 30 min of stabilization at room temperature, the sections were washed and incubated with blocking solution (3% BSA, 0.1% Tween-20, and 5% goat serum in PBS) for 2 h at room temperature. Next, the sections were washed with PBS and incubated overnight at 4˚C with Cy3 secondary antibody goat anti-mouse IgG + IgM (1:200; Jackson ImmunoResearch Europe Ltd., 115-165-044) diluted in a solution of 1% BSA in PBS. The following day, the sections were washed in PBS and mounted on Superfrost microscopy slides (Thermo Fisher Scientific Inc.). Sections were embedded using Vectashield with DAPI (Vector Labs) and sealed with a cover glass and nail polish. Slides were left to dry for 20 min at room temperature followed by storage at 4˚C until imaging the next day.

For cell type staining, only tissue of intervention animals was used. Sections between + 0.7 and − 1.1 mm Bregma were used due to its central location in the brain. Sections were stabilized at room temperature for 30 min, followed by a washing step and incubation with blocking solution (3% BSA, 0.1% Tween-20, and 5% goat serum in PBS) for 2 h at room temperature. The sections were washed with PBS, and incubated with either the rabbit anti mouse Iba1 primary antibody (1:500; FUJIFILM Wako Pure Chemical Corporation, 019-19741), the rabbit anti mouse NeuN primary antibody (1:200; Thermo Fisher Scientific Inc., 702022), or the chicken anti mouse GFAP primary antibody (1:200; Thermo Fisher Scientific Inc., PA1-10004) diluted in the beforementioned blocking solution overnight at 4˚C. The next day, the sections were washed with PBS and incubated with both Cy3 secondary antibody goat anti-mouse IgG + IgM (1:200; Jackson ImmunoResearch Europe Ltd., 115-165-044) and either Cy5 goat anti rabbit secondary antibody (1:500; Thermo Fisher Scientific Inc., A10523) or Alexa Fluor 647 goat anti chicken secondary antibody (1:200; Invitrogen, A21449), diluted in a solution of 1% BSA in PBS overnight at 4˚C. On the third day, the sections were washed with PBS and mounted on Superfrost microscopy slides (Thermo Fisher Scientific Inc.). Vectashield with DAPI (Vector Labs) was added to embed the sections, and the slide was sealed with a cover glass and nail polish. Slides were left to dry for 30 min at room temperature followed by storage at 4˚C until imaging.

### Confocal imaging

All images were taken using a Stellaris 5 confocal microscope (Leica Microsystems).

Per mouse, 1 tile scan was made for a whole brain section to image the DAPI and Ig signal. Images were made with a 10 × 0.40 NA dry objective at a resolution of 1024 × 1024 (zoom factor = 4.13, speed = 600 Hz, bidirectional). Focus points were manually set to control for uneven z-positions in the section. The laser powers and detector gain values were set while minimizing the overexposure at an Ig hotspot. These set values were consistently used for all the imaged sections, and all the sections were imaged on the day after the staining.

Additional z-stacks were made of the hotspots in the mice infused with lectin in order to identify the vessel type where the leakage possibly originated from. The z-stacks were imaged using a 40 × 1.3 NA oil objective at a resolution of 1024 × 1024 (zoom factor = 1.0, speed = 400 Hz, bidirectional, step size = 1 μm).

For imaging of the cell types, z-stack tile scans (290.63 × 290.63 μm) of approximately 10 μm depth were made of 2 hotspots per section using a 40 × 1.3 NA oil objective at a resolution of 1024 × 1024 (speed = 200 Hz, bidirectional, step size = 0.24 μm, line averaging = 2). The selection of the hotspots was done using visual inspection based on the brightest hotspots in the section in combination with the detection of signal from the stained cell type.

### Quantifications

All quantifications were done in Image J Fiji software version 1.54p.

One coronal section per animal was used for quantification of the Ig signal. To quantify the total Ig signal, a region of interest (ROI) was manually delineated around the entire section, excluding the ventricles. Within this ROI, the mean grey value was calculated and is referred to as the mean signal intensity. For the segmentation, 8 brain areas were segmented bilaterally according to the Allen Coronal Mouse Brain Atlas. Areas included the cortex, corpus callosum, hippocampus, fimbria, thalamus, hypothalamus, amygdala, and the basal ganglia which include a subset of areas including the caudoputamen, globus pallidus, internal capsule, and the substantia innominate. The mean signal intensity was calculated for each of these areas after manually drawing a ROI around the segment. Three out of the 144 brain regions were absent or only partially present due to tissue damage and these were excluded and recorded as missing values. To estimate the size of each leakage hotspot, two lines were drawn, one horizontal and one vertical, across the hotspot. The intensity profiles along these lines were fitted with Gaussian functions, from which the full width at half maximum (FWHM) was determined. For each brain, the median FWHM across all hotspots was calculated. To approximate the 3D FWHM of the hotspots, the 2D median FWHM was scaled by a factor of 4/π, correcting for the fact that the measurements were taken from 2D sections intersecting 3D structures at random orientations.

The imaged hotspot z-stacks with lectin signal were visually inspected to assess the presence of a lectin positive blood vessel in the middle of the hotspot. If present, the size of the vessel lumen was calculated by measuring the width of the lectin signal from the maximum intensity projection of the z-stack.

To assess interactions between Ig and specific cell types, z-stack tile scan images of hotspot regions were analyzed. In each hotspot, approximately 10 ROIs were drawn around cells exhibiting enhanced Ig signal near the nucleus. The Ig signal within each ROI was visually compared to the corresponding cell-type marker channel to determine the presence or absence of that cell type. To reduce background noise, Otsu thresholding was applied to the cell-type channels in ImageJ. Interactions were defined as overlap between Ig and thresholded Iba1 or GFAP signals for microglia and astrocytes respectively. For neurons, interaction was scored when Ig signal surrounded the NeuN-positive nucleus, as NeuN labels the nucleus rather than the cytoplasm. For each animal and cell type, an interaction ratio was calculated by dividing the number of ROIs showing interactions by the total number of ROIs assessed. One animal was excluded from the microglia analysis and two animals from the astrocyte analysis due to absence of Ig-positive cells or cell-type marker signal in hotspot regions.

To account for differences in cell-type abundance within the assessed hotspot tile scans, Ig interaction ratios were normalized to the relative abundance of each cell type. First, the mean number of Iba1-, GFAP-, and NeuN-positive cells per tile scan was calculated across all assessed hotspot tile scans from all intervention animals by manual counting. A cell-type abundance ratio was then determined for each cell type by dividing the mean number of that cell type by the sum of the mean numbers of all assessed cell types. Subsequently, for each animal and cell type, the Ig interaction ratio was divided by the corresponding cell-type abundance ratio to obtain a normalized measure of relative Ig interaction.

### Statistical analyses

All statistical analyses were performed in GraphPad Prism 10 software. Significance was tested using α < 0.05.

To compare the mean Ig signal of the control and intervention group, a Welch’s t-test was performed as the data was normally distributed, but no equal variance was assumed.

For the segmentation data, a mixed-effects model was used with a Geisser-Greenhouse correction as sphericity was not assumed. A Tukey multiple comparisons test was performed to compare the means of the control and intervention group per brain area, and to compare the means of the different brain areas per group.

Differences in Ig interaction ratios and relative Ig interactions for the different cell types were statistically tested using the Welch’s ANOVA with a Dunnett’s T3 multiple comparisons test as the data appeared to be normally distributed but not equal in variance.

## Results

### Evans blue extravasation

**Fig. 2:**
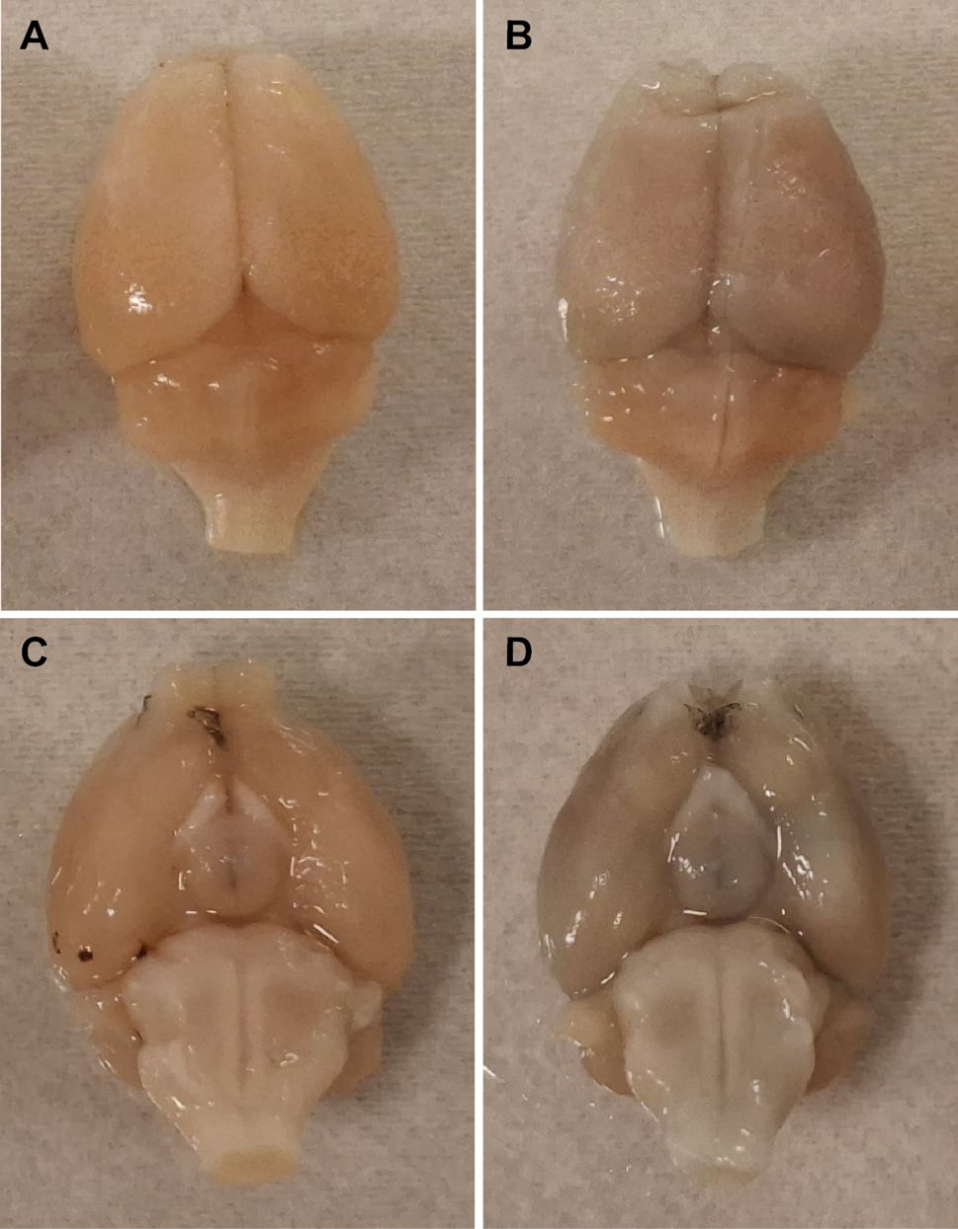
Typical example of Evans blue extravasation upon the use of PDUS combined with MBs. (**A**) Top-view and (**C**) bottom-view images of a control mouse brain, and (**B**) top-view and (**D**) bottom-view images of an intervention mouse brain injected with EB dye

Macroscopic examination of brains infused with EB revealed that the brains retrieved from intervention mice that received both the PDUS and MBs exhibited markedly blue coloration compared to those from control animals that received PDUS and PBS, visible at both the dorsal and ventral part of the cerebrum (Fig. 2). This enhanced blue coloration suggests widespread EB extravasation, which appeared to be distributed across the entire surface of the cerebrum.

### Immunoglobulin leakage

**Fig. 3:**
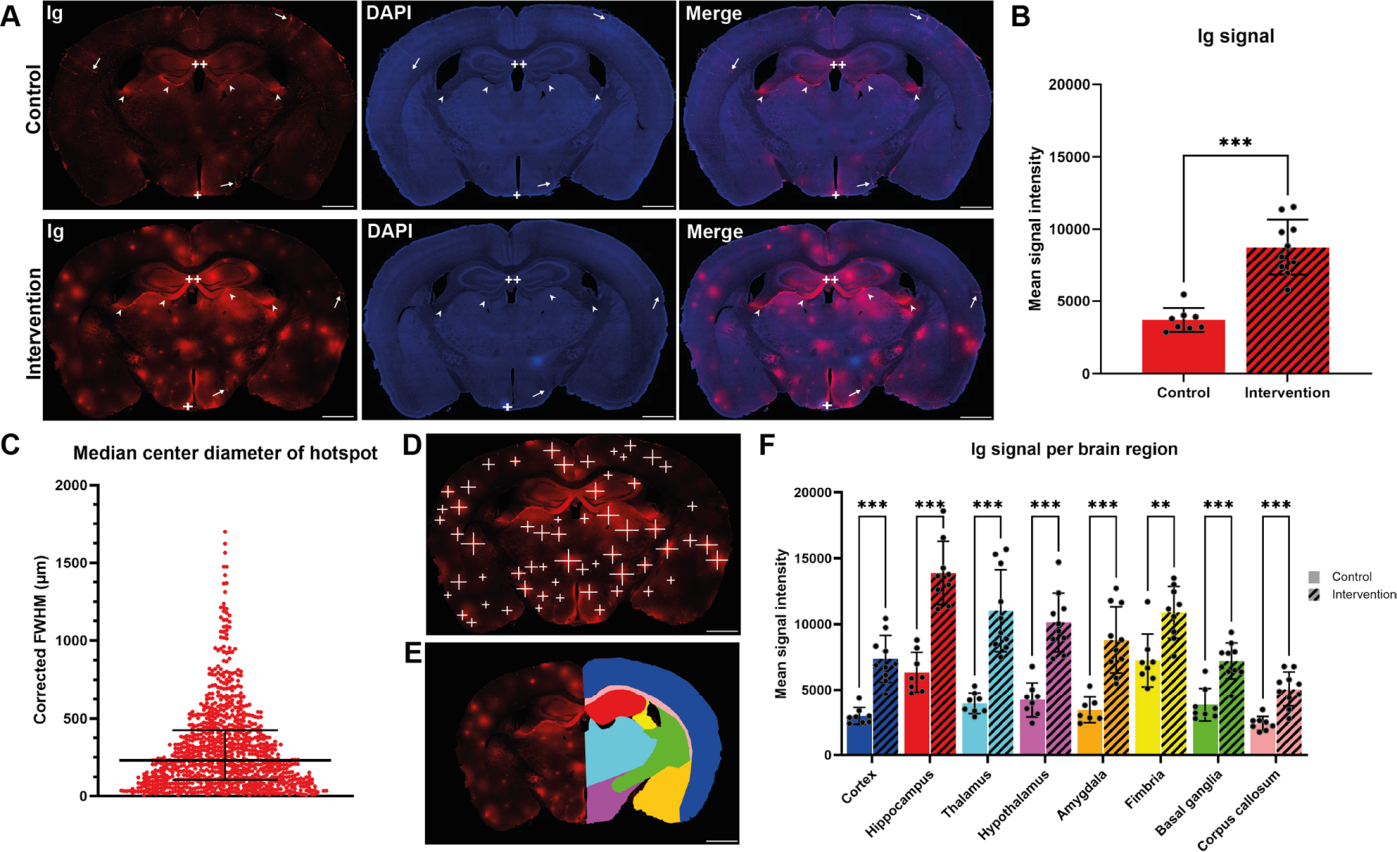
Confocal images and quantifications of immunoglobulins in coronal mouse brain sections following PDUS combined with MBs. (**A**) Representative microscopic images of coronal mouse brain sections of control (top row) and intervention (bottom row) mice stained for Ig. Arrows represent enhanced Ig signal around penetrating vessels, arrowheads represent Ig signal at the border between the thalamus and hippocampus or fimbria, + represents enhanced Ig signal at the median eminence, and + + represent enhanced Ig signal at the medial parts of the hippocampus. Scale bars represent 1 mm. (**B**) Mean signal intensity of the Ig signal for both the control and intervention group. Bars and error bars are shown as mean ± SD. (**C**) The median center diameter of all the hotspots based on the corrected FWHM. Data is shown as median ± IQR. (**D**) Example image of the line placements in a tissue section to calculate the center diameter of the hotspots. (**E**) Example of unilateral ROI placement used for segmentation analysis; full analysis was performed bilaterally. The segmentation colors correspond to the colors of the regions shown in graph (**F**). (**F**) Mean signal intensity of the Ig signal for the segmented brain regions of both the control and intervention group. Bars and error bars are shown as mean ± SD. ** = *adjusted p* < 0.01, *** = *adjusted p* < 0.001

In controls, Ig localization was restricted to specific regions including the medial hippocampi, the hypothalamic area around the third ventricle (including the median eminence, a circumventricular organ), larger penetrating or parenchymal blood vessels, and the borders between the thalamus and hippocampus as well as the thalamus and fimbria (Fig. 3A, top row). The intervention group exhibited this same regional pattern, with additional Ig signal hotspots distributed broadly throughout the brain sections (Fig. 3A, bottom row). Quantitative analysis confirmed a significantly higher mean Ig fluorescence in the intervention group (*n* = 10) compared to controls (*n* = 8) (one-tailed Welch’s t-test, *t*(12.80) = 7.53, *p* < 0.001) (Fig. 3B).

Segmentation analysis was performed on the same images using a linear mixed-effects model (with Geisser-Greenhouse correction), and revealed significant main effects of group (*F* [1, 16] = 47.07, *p* < 0.001), brain region (*F*(3.23, 50.23) = 59.05, *p* < 0.001), and a significant group × brain region interaction (*F*(7, 109) = 11.06, *p* < 0.001). Post hoc Tukey’s multiple comparisons tests showed that all eight brain regions in the intervention group had significantly higher Ig signal intensities than their corresponding regions in the control group (*all adjusted p* < 0.01; Fig. 3E, F), suggesting a consistent increase in BBB permeability in all the analyzed brain regions. However, although the corpus callosum of the intervention group exhibited significantly elevated Ig signal relative to the control group (*adjusted p* < 0.001), this signal was significantly lower than that of the other brain regions (*adjusted p* < 0.001 for all comparisons), indicating a comparatively lower degree of BBB opening in this region. Additionally, in the control group, both the hippocampus and fimbria displayed significantly higher Ig signals compared to the other brain regions (*adjusted p* < 0.05 for all comparisons), consistent with the region-specific Ig pattern previously noted.

### Leakage hotspot assessment

Altogether, 902 line profiles of Ig hotspots were analyzed from all the intervention mice (*n* = 10). The median diameter of the hotspots (corrected FWHM) was calculated to be 230.7 μm (IQR = 105.3–426.4 μm) (Fig. 3C, D).

**Fig. 4:**
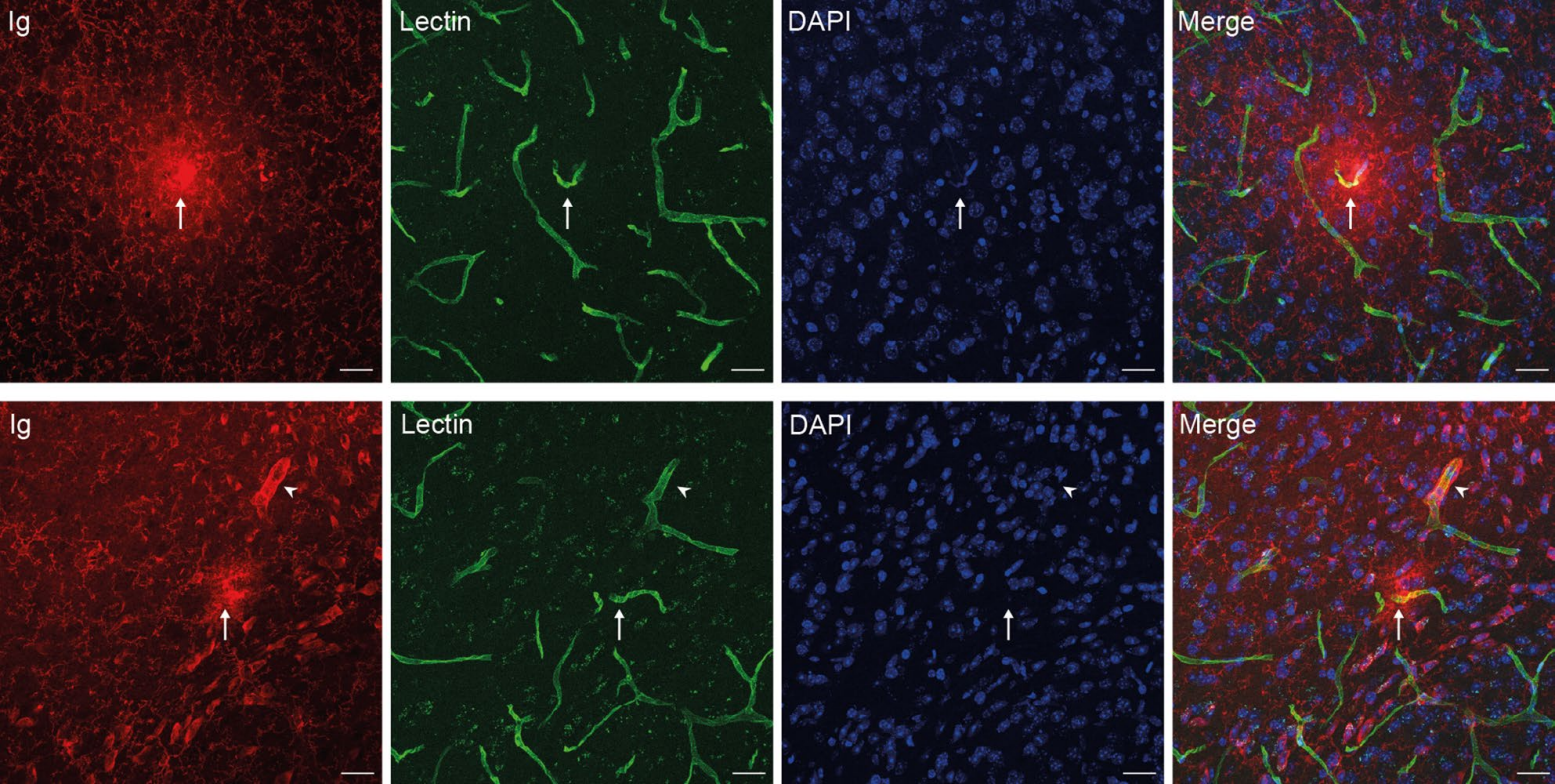
Representative microscopic images showing possible vascular origins of immunoglobulin leakage hotspots in an intervention mouse. Top and bottom row show two distinct Ig leakage hotspots. Ig marks the presents of extravasated Igs, lectin labels the vessels intravascularly, and DAPI labels the nuclei. Both rows show a lectin positive vessel in the middle of an Ig hotspot, indicated with an arrow to show the possible vascular origin of the leakage. In the bottom row, an additional lectin positive vessel with enhanced Ig signal was found near the leakage hotspot, indicated with an arrowhead. Scale bars represent 25 μm

For identification of the leakage vessel type, 11 of the 28 inspected hotspots showed a lectin positive vessel in the middle of the hotspot which appeared to be the origin of the leakage (Fig. 4). The lumen diameter of these blood vessels was between 5 and 6 μm, indicating that these are capillaries. Occasionally, a blood vessel located near a leakage hotspot showed enhanced Ig signal surrounding the vessel lumen without a diffusion gradient, unlike the Ig hotspots (Fig. 4, bottom row, arrowhead).

### Identification of parenchymal cell types interacting with immunoglobulins

**Fig. 5:**
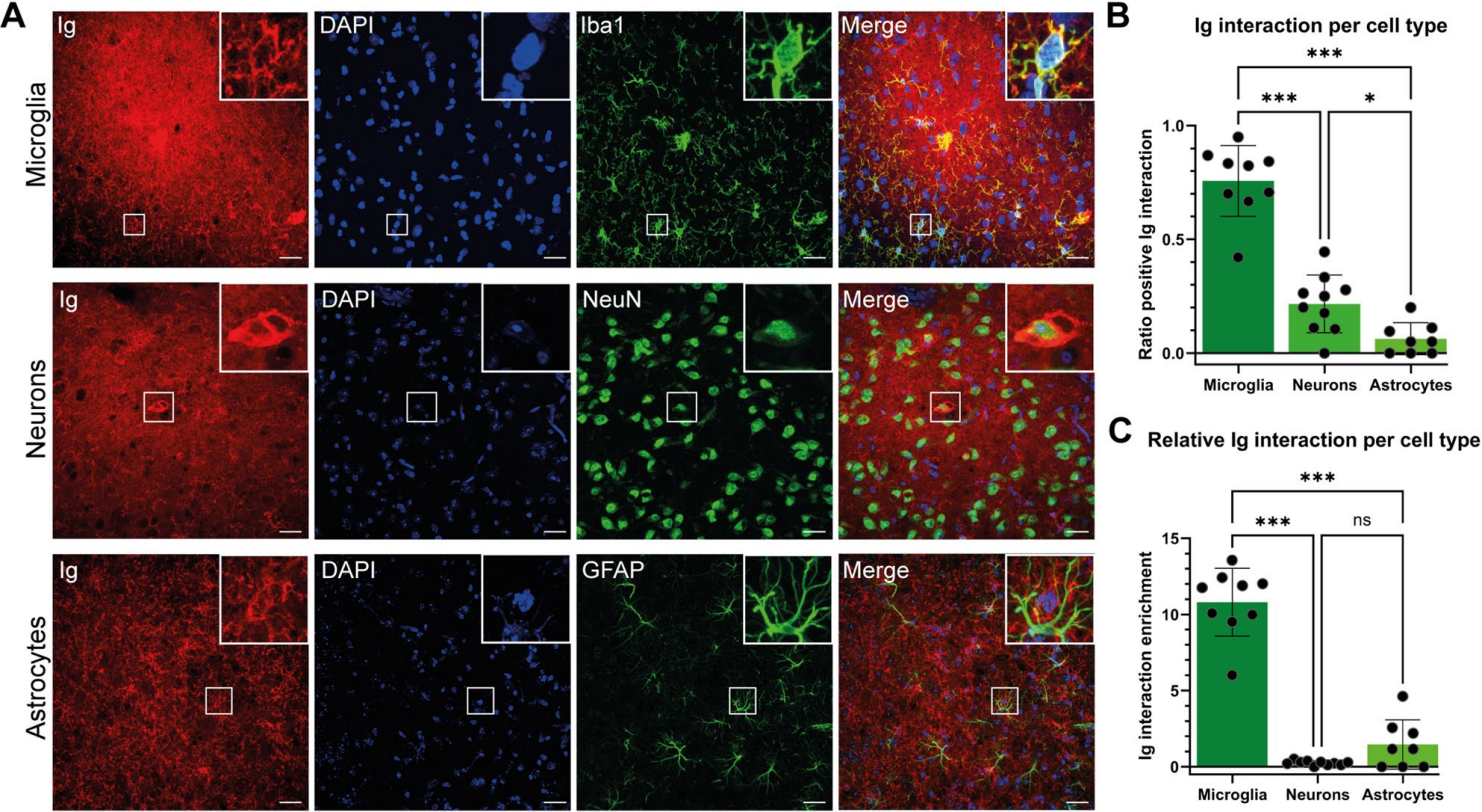
Identification of cell-types interacting with immunoglobulin in leakage hotspots after PDUS combined with MBs. (**A**) Zoomed-in microscopic images of leakage hotspots, co-stained for Ig (*red*), DAPI (*blue*), and either Iba1 (*green*; top row) for microglia, NeuN (*green*; middle row) for neurons, or GFAP (*green*; bottom row) for astrocytes. A squared region of interest is shown which presents a cell with enhanced Ig signal. In these examples, positive interactions of Ig with microglia and neurons are shown, and a negative interaction of Ig with astrocytes. Scale bars represent 25 μm. (**B**) Ratio of Ig-positive interactions per cell type. Each data point represents the interaction ratio of an individual animal. Bars and error bars indicate mean ± SD. (**C**) Cell type-normalized Ig interaction. The interaction ratio per cell type was normalized to the relative abundance of that cell type within the assessed tissue to account for differences in cell-type prevalence. Each data point represents the normalized interaction ratio of an individual animal. Bars and error bars indicate mean ± SD. ns = not significant, * = *p* < 0 0.05, ** = *p* < 0.01, and *** = *p* < 0.001

We noted that cells in the vicinity of a leakage site frequently interacted with the Igs. Immunostainings were done to identify these cells. A Welch’s ANOVA with Dunnett’s T3 multiple comparisons test showed significant differences in Ig interaction ratios among microglia, neurons, and astrocytes in all intervention mice samples (*n* = 10) (Welch’s *F*(2.00, 15.04) = 69.68, *p <* 0.001). Microglia exhibited the highest Ig interaction ratio of 76% ± 16% (mean ± SD), which was significantly greater than that of neurons (22% ± 13%, *adjusted p* < 0.001) and astrocytes (6% ± 7%, *adjusted p* < 0.001) (Fig. 5B). Neurons also had a significantly higher ratio than astrocytes (*adjusted p* = 0.017).

Because cell-type abundance differed substantially within the assessed regions (7% microglia, 88% neurons, and 4% astrocytes), interaction ratios were additionally normalized to cell-type abundance to assess relative Ig interaction enrichment. A Welch’s ANOVA with Dunnett’s T3 multiple comparisons test again demonstrated significant differences among cell types (*n* = 10) (Welch’s *F*(2.00, 10.06) = 96.73, *p <* 0.001). Microglia showed the highest Ig interaction enrichment (10.8 ± 2.2, mean ± SD), which was significantly greater than that of neurons (0.24 ± 0.14, *adjusted p <* 0.001) and astrocytes (1.5 ± 1.6, *adjusted p <* 0.001) (Fig. 5C). After normalization for cell-type abundance, no significant difference in Ig interaction enrichment was observed between neurons and astrocytes (*adjusted p =* 0.183). Examples of positive interactions of Ig signal with microglia and neurons, and an absence of interaction with astrocytes, are shown in Fig. 5A.

### Structural integrity of blood vessels

**Fig. 6:**
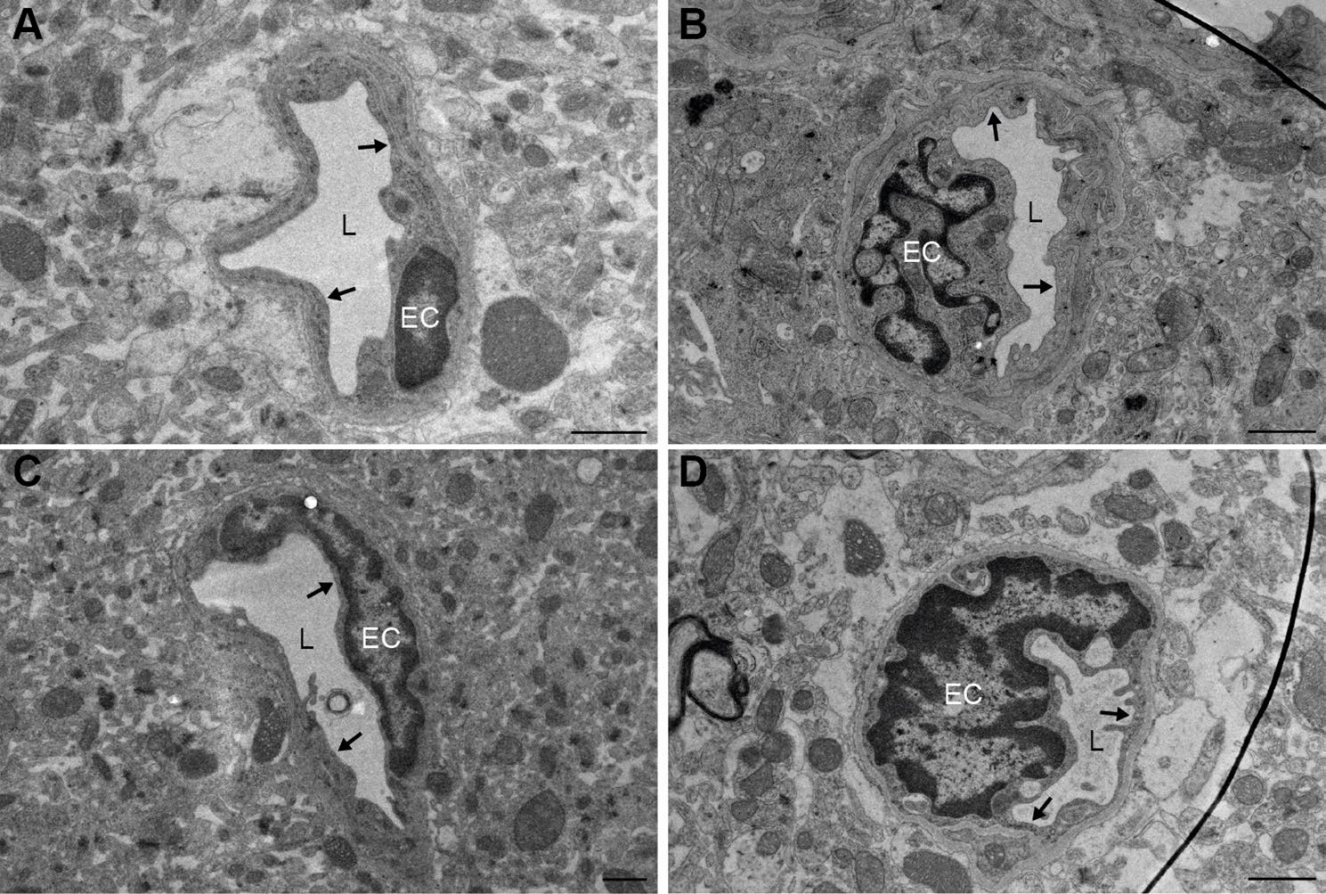
Representative micrographs of cerebral blood vessels after PDUS combined with MBs. Images of blood vessels from both control (**A**,** C**) and intervention (**B**,** D**) mice. There are no erythrocyte extravasations visible, nor perforations in the endothelial lining of the vessel wall as indicated using arrows. L = lumen, EC = endothelial cell nucleus. Scale bar represents 1 μm

Visual inspection of TEM images revealed no extravasated erythrocytes outside of the blood vessels, nor any perforations in the endothelial lining of the vessel wall in any of the 69 blood vessels imaged from the intervention group (*n* = 4) or the 66 vessels from the control group (*n* = 4) (Fig. 6), indicating that the BBB opening in the intervention group did not cause overt structural damage to the vessels.

## Discussion

In this study, we demonstrate that PDUS combined with MBs reliably induces widespread and non-destructive BBB opening in mice. This was confirmed by EB extravasation across the cerebrum, and by IF showing leakage of endogenous Igs into the brain parenchyma. TEM revealed no overt structural damage to the vasculature. Vessel walls remained intact and no erythrocyte extravasation was observed, suggesting the absence of inertial cavitation events within the exposed cerebral vessels.

Ig distribution in control animals showed endogenous presence in specific brain areas, including the medial hippocampi, the hypothalamus (particularly surrounding the third ventricle and median eminence) and around large penetrating vessels. Similar findings were reported by Yoshimi et al., who described diffuse IgG staining in comparable regions of the intact mouse brain [24]. The signal observed near the median eminence is consistent with its known lack of a tight BBB, as it is a circumventricular organ [25]. Ig presence in other regions, such as the hippocampal–thalamic borders and large penetrating vessels, may reflect proximity to CSF spaces which physiologically contain Igs [26]. Yoshimi et al. further speculated that diffuse IgG staining may originate from leakage at the level of the choroid plexus, pia mater, or the BBB itself [24]. However, reports describing endogenous Ig localization in the intact brain remain limited, and both its origin and functional significance warrant further investigation.

Following BBB opening, Ig leakage was not uniformly distributed but appeared in discrete, regionally localized hotspots throughout the brain. This non-uniform pattern suggests that BBB permeability changes occur at specific locations, potentially influenced by local vascular properties, MB dynamics or simply by chance. The secondary antibody that we used to identify Ig leakage recognizes both IgG (~ 150 kDa) and IgM (~ 900 kDa) proteins [26]. Given the substantially larger size and lower serum concentration of IgM (0.8–1.46 g/L) compared to IgG (6.08–15.15 g/L) [27], it is likely that the majority of the observed leakage signal originates from IgG. The relatively large molecular size of IgG may explain the formation of discrete leakage hotspots, as only regions with more substantial BBB disruption would permit its extravasation. In contrast, smaller molecules may have crossed the BBB from a larger number of smaller disruption sites, but would remain undetected using Ig-based staining. Additionally, the larger size of IgG may limit its diffusion within the parenchyma, resulting in a more restricted spread from the initial leakage site compared to smaller compounds. This is further substantiated by the more diffuse extravasation pattern of EB, which has a smaller molecular weight (69 kDa when bound to albumin) as compared to IgG [28].

Notably, the corpus callosum showed less Ig leakage compared to gray matter regions, likely due to its lower vascular density [29]. Most leakage appeared centered around capillary-sized vessels, suggesting that smaller vessels are more susceptible to PDUS-induced BBB opening. This may be attributed to the greater mechanical forces exerted during MB cavitation within narrower lumens. Supporting this, previous research using FUS has demonstrated that smaller MBs in larger vessels require higher acoustic pressures to achieve comparable levels of cavitation, indicating that cavitation effects may be amplified in smaller vessels under standard PDUS conditions [30].

Additionally, we occasionally observed blood vessels near Ig leakage hotspots that exhibited enhanced Ig signal confined to the vessel wall or immediate perivascular region, without a surrounding diffusion gradient. This pattern may reflect the presence of Igs within endothelial cells or CSF-filled perivascular spaces. In the context of BBB disruption, endothelial cells may mediate the clearance of endogenous IgG from the parenchyma to the circulation through endocytosis facilitated by the neonatal Fc receptor (FcRn) [31]. Furthermore, perivascular spaces may act as drainage pathways for extravasated Igs, facilitating its clearance from the brain parenchyma and resulting in perivascular signal accumulation. A similar observation was reported by Georgakopoulou et al. [32] who found increased IgG signal in perivascular spaces of vessels located near microinfarcts. These microinfarcts were characterized by IgG leakage around fluorescent microspheres lodged within the vasculature, showing a similar pattern of IgG leakage as the Ig hotspots shown in the present study.

To assess whether the observed IgG leakage spread from the vasculature could be explained by passive diffusion, we calculated the theoretical diffusion distance using the equation x = sqrt(2Dt), where D is the diffusion coefficient and t the diffusion time. Using an in vivo tissue-derived diffusion coefficient for IgG (D = 6.42 × 10^− 8^ cm²/s) [33] and assuming an average diffusion time of 35 min (half of the 70 min BBB opening period), the theoretical diffusion distance was estimated at approximately 164 μm. In comparison, the observed spread from the vessel of origin was ~ 115.5 μm, based on half the measured FWHM of the Ig hotspots. These values fall within the same order of magnitude, suggesting that the pattern of Ig leakage may be explained by passive diffusion.

In Ig leakage hotspots, we observed Ig signals in close association with surrounding cells. These cells appeared to be predominantly microglia, with a smaller contribution from neurons and astrocytes. Both humans and mice possess microglia that express Fcγ receptors capable of binding IgG, triggering either pro-inflammatory or anti-inflammatory responses depending on the receptor subtype [34]. This likely explains the prominent microglial involvement observed in our data. However, some neurons and astrocytes may have gone undetected due to limited NeuN and GFAP expression in certain subtypes [35, 36] which could lead to underestimation of neuronal and astrocytic Ig interaction.

While our findings support the utility of PDUS combined with MBs as a BBB dysfunction model, there are limitations. First, the IF detection of Ig targeted both IgG and IgM, limiting size-selectivity conclusions. Second, the use of specific cell markers (Iba1, NeuN, GFAP) may not capture all relevant cell subtypes. Lastly, while TEM showed no structural vessel damage, sampling bias cannot be excluded as the examined sections may not have overlapped with leakage sites. EB-based region selection was aimed to minimize this risk, but uncertainty remains. To account for these limitations, future studies should define the size-selectivity of the BBB opening to pathologically relevant sizes (e.g. fibrinogen, thrombin, albumin, IgG, and iron) as found in Alzheimer’s disease brains [4], and assess potential vascular damage while reducing possible sampling bias by using complementary histological approaches such as hematoxylin and eosin staining for erythrocytes in larger tissue sections.

## Conclusions

PDUS combined with microbubbles presents a promising approach for establishing an inducible and controlled BBB dysfunction model, as it consistently induces widespread and non-destructive leakage across the brain. Although certain characteristics of the model require further clarification, including the timing of BBB opening, the size-selectivity of the disruption, and its overall safety profile, it offers a valuable platform for studying the direct consequences of BBB permeability changes both in vivo and post-mortem. The in vivo component is especially advantageous, as it enables the investigation of BBB dysfunction within a physiologically relevant setting where systemic and neural processes interact. Beyond its use as a research tool, this approach may also hold potential for facilitating brain-wide delivery of therapeutic agents in future applications. Finally, the model enables the study of post-leakage processes such as blood component clearance, neuroinflammation, and neurotoxicity, which are critical in the context of aging and neurodegenerative diseases.

## Supplementary Information

Below is the link to the electronic supplementary material.

Supplementary Material 1

## Data Availability

All data generated or analysed during this study are included in this published article.

## Abbreviations

BBB: Blood-brain barrier
CSF: Cerebrospinal fluid
DAPI: 4′,6-diamidino-2-fenylindool
EB: Evans blue
EC: Endothelial cell
FWHM: Full width at half maximum
FcRn: Neonatal Fc receptor
FUS: Focused ultrasound
GA: Glutaraldehyde
GFAP: Glial fibrillary acidic protein
Iba1: Ionized calcium-binding adapter molecule 1
IF: Immunofluorescence
Ig: Immunoglobulin
IQR: Interquartile range
MB: Microbubble
NeuN: Neuronal nuclei
PBCA: Poly(butyl cyanoacrylate)
PBS: Phosphate buffer saline
PDUS: Power Doppler ultrasound
PFA: Paraformaldehyde
ROI: Region of interest
SD: Standard deviation
TEM: Transmission electron microscopy
US: Ultrasound
