# Supplementary material for: Induction of subtle blood-brain barrier dysfunction using preclinical diagnostic ultrasound combined with microbubbles
Source: Fluids Barriers CNS. 2026 Jun 2;23:78. doi: 10.1186/s12987-026-00820-7 (PMC13231714; doi:10.1186/s12987-026-00820-7)
Supplement: Supplementary file 1 — Supplementary Material 1 [file 12987_2026_820_MOESM1_ESM.docx]

***Supplementary material***

**Induction of subtle blood-brain barrier dysfunction using preclinical diagnostic ultrasound combined with microbubbles**

*Shakira A. van der Panne^1,2,3^, Isabella Z. Koster^1^, Anita E. Grootemaat^4^, Nicole N. van der Wel^4^, Mario G. Ries^5^, Helga E. de Vries^2,6^, Louise van der Weerd^7,8^, Gustav J. Strijkers^1,3^, Erik N.T.P. Bakker^1, 2,3^*

^1^Department of Biomedical Engineering and Physics, Amsterdam University Medical Center, Amsterdam, the Netherlands

^2^Amsterdam Neuroscience, Amsterdam University Medical Center, Amsterdam, the Netherlands

^3^Amsterdam Cardiovascular Sciences, Amsterdam University Medical Center, Amsterdam, the Netherlands

^4^Electron Microscopy Center Amsterdam, Amsterdam University Medical Center, Amsterdam, the Netherlands

^5^Center for Imaging Sciences, University Medical Center Utrecht, Utrecht, the Netherlands

^6^Department of Molecular Cell Biology and Immunology, Amsterdam University Medical Center, Amsterdam, the Netherlands

^7^Department of Radiology, Leiden University Medical Center, Leiden, the Netherlands

^8^Department of Human Genetics, Leiden University Medical Center, Leiden, the Netherlands

**Corresponding author:** Shakira A. van der Panne

**Supplementary Figure S1**


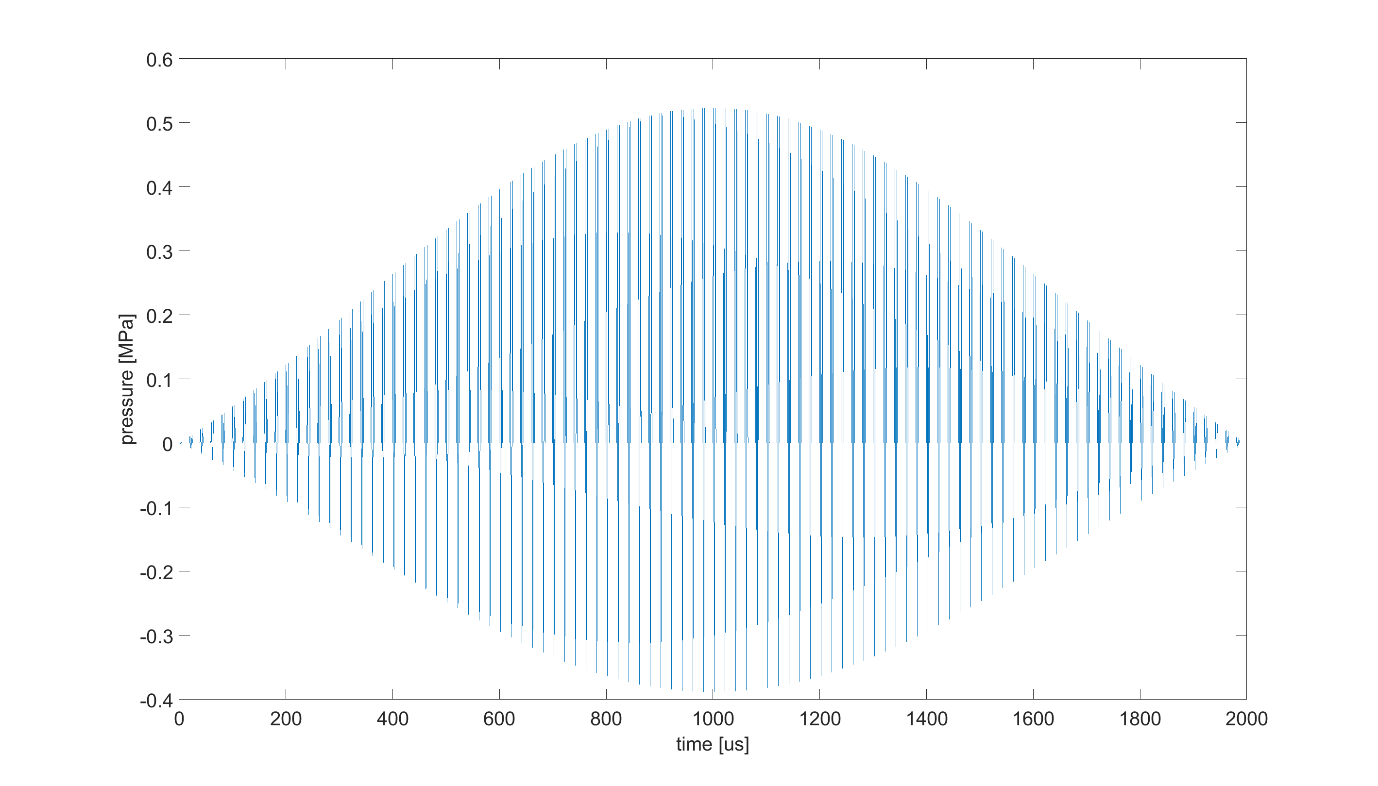


**Supplementary Figure S1: Schematic representation of the effective burst pulse train of the acoustic exposure.** Each burst consists of 100 pulses which in turn consist of a 1.5-cycle 12.5MHz signal repeated with a PRF of 50kHz. The entire burst pulse is repeated with 40Hz.

**Supplementary Methods**

As neither the details of the ultrasound imaging sequence nor the exact acoustic specifications of the employed transducer are disclosed by FUJIFILM VisualSonics Inc., the overall acoustic exposure was experimentally characterized.

To this end, a calibrated Precision Acoustics fiber optic hydrophone was used in combination with a Keysight DSO2022 oscilloscope, synchronized with the ultrasound acquisition. The acoustic waveform and pressure amplitudes were evaluated both in water and intracranially using the cranium of a freshly sacrificed mouse cadaver of comparable phenotype and age. For intracranial measurements, a small stainless steel guide tube was inserted through the base of the skull. The position of the tube was verified using ultrasound imaging, after which the hydrophone measurement fiber was inserted through the guide tube. This approach enabled intracranial measurements of acoustic exposure without altering the superior ultrasound beam path.

The following acoustic parameters were measured:
• the overall waveform of the acoustic exposure,
• the acoustic pressure of each pulse, including peak negative pressure (PNP) and peak to peak pressure (PTP), and
• the frequency specific attenuation of the mouse cranium at 12.5 MHz.


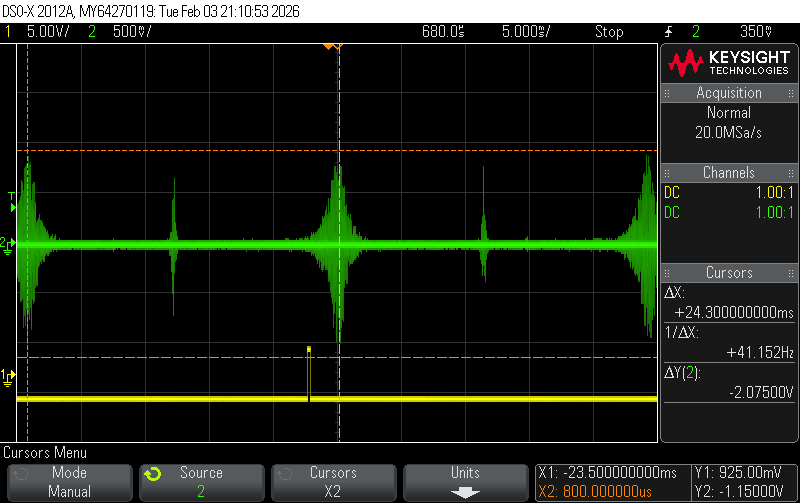


Example image of the overall waveform of the imaging sequence measured in water. Visible in the center is the pulse train of the power Doppler sequence, which repeats with 40Hz frame-rate.


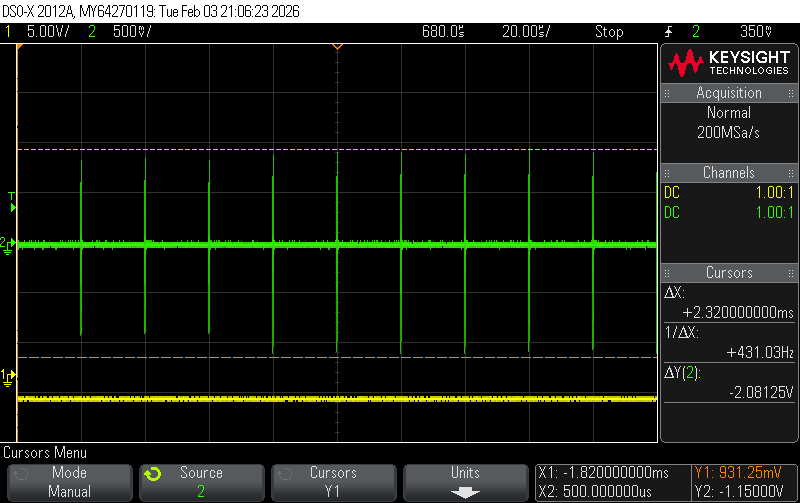


Zoom-in on the center imaging pulse train (50kHz PRF) displaying the spacing of the excitation pulses of the doppler imaging sequence. Noteworthy is hereby that each element fires thereby twice (most likely due to compounding of the imaging sequence).


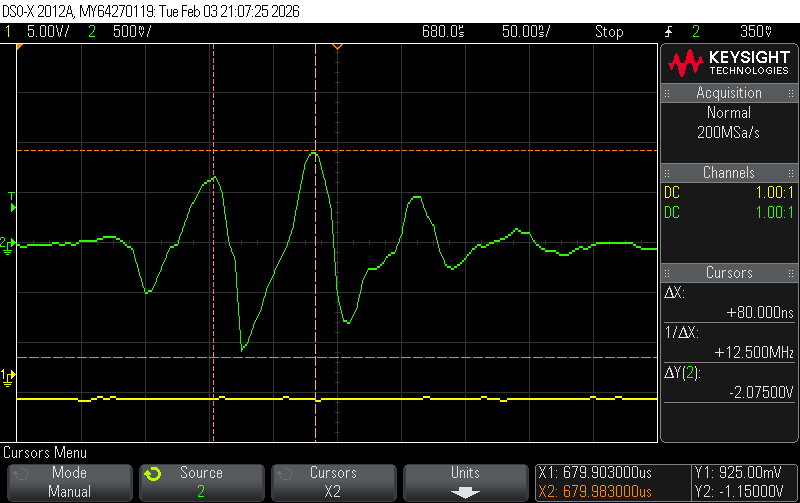


And a zoom-in on one of the excitation pulses (1.5-cycle 12.5MHz) itself. The slightly prolonged oscillation is typical for image transducer ringing.


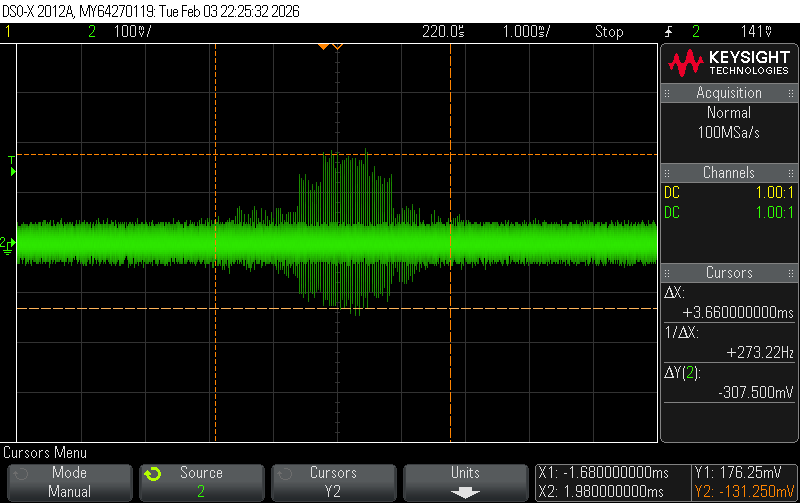


And finally the pulse train measured invasively in a cadaver mouse brain near the ventricle. The cranial pressure attenuation at 12.5MHz was measured as 14.9dB (i.e. 18% of the acoustic pressure compared to a free-water measurement).

The acoustic amplitude varied very little in superior inferior direction over the dimension of the mouse brain (due to the linear transducer geometry), and in LR-direction about 20%.
